# Supplementary material for: Genetic risk factors for ME/CFS identified using combinatorial analysis
Source: J Transl Med. 2022 Dec 14;20:598. doi: 10.1186/s12967-022-03815-8 (PMC9749644; doi:10.1186/s12967-022-03815-8)
Supplement: Supplementary file 1 — Additional file 1: Supplemental data from the analyses described in the paper. [file 12967_2022_3815_MOESM1_ESM.docx]

**Supplementary Data:**

**Genetic Risk Factors for ME/CFS Identified using Combinatorial Analysis**

**Author(s): Sayoni Das, Krystyna Taylor, James Kozubek, Jason Sardell, Steve Gardner**

**Date:** 5^th^ December 2022

# Supplementary Data

## Pain Questionnaire Study Design

UK Biobank participants included in the Pain Questionnaire case cohort answered positively to the question:

| Data Type | ICD-10 / Data Field ID | Description |
| --- | --- | --- |
| Self-reported | 120010 | Ever had Chronic Fatigue Syndrome or Myalgic Encephalitis (M.E.) |

UK Biobank participants excluded from the Pain Questionnaire control cohort met one or more of the following criteria:

Table 5: Control selection criteria for the ME/CFS Pain Questionnaire cohort

| Data Type | ICD-10 / Data Field ID | Description |
| --- | --- | --- |
| HES | G93.3 | Postviral fatigue syndrome |
| HES | R53.x | Malaise and fatigue |
| HES | G47.x | Sleep disorders |
| HES | M79.7 | Fibromyalgia |
| Self-reported | 120009 | Ever had fibromyalgia syndrome? |
| Self-reported | 120010 | Ever had Chronic Fatigue Syndrome or Myalgic Encephalitis? |
| Self-reported | 120120 | Exercise brings on fatigue |
| Self-reported | 120122 | Fatigue interferes with physical functioning |
| Self-reported | 120123 | Fatigue causes frequent problems |
| Self-reported | 120124 | Fatigue prevents sustained physical functioning |
| Self-reported | 120125 | Fatigue interferes with carrying out certain duties or responsibilities |
| Self-reported | 120126 | Fatigue is among three most disabling symptoms |
| Self-reported | 120127 | Fatigue interferes with work, family or social life |

### Genotype Quality Control

Appropriate quality control of genotype data was performed using GRAF^39^ (Genetic Relationship and Fingerprinting) and PLINK^40^ based on standard quality control procedures to ensure thorough cleaning of the data before it is used for genomic analyses.

This included the following steps:

1. Batch effect correction: Batch-level quality control procedures was performed based on recommendations by UK Biobank^32^ and only SNPs that pass all batch-level QC were used for further analysis.
2. Sample and SNP filtering based on missingness: The filtering for SNPs with missing data (<5%) was followed by filtering of individuals with missing data (<5%) using PLINK.
3. Minor Allele Frequency (MAF) filtering of SNPs: The genotype data would be filtered to exclude SNPs with MAF <0.0001 using PLINK.
4. Hardy-Weinberg Equilibrium (HWE) filtering: HWE filtering was performed on controls with *p*<10^-10^ using PLINK.
5. Heterozygosity filtering: Samples with extreme (very high or very low) heterozygosity were removed.
6. Sample filtering based on relatedness: GRAF-rel^39^ was used to identify duplicates and closely related subjects in the dataset. After identification of close relatives, only one representative of each closely related family pairs was retained.
7. Ancestry analysis: GRAF-pop was used for ancestry inference and limit samples for the dataset to European ancestry.
8. Sex discrepancy of individuals: Samples that have discrepancies between the sex recorded in the dataset and their sex based on absence/presence of a Y chromosome were removed.


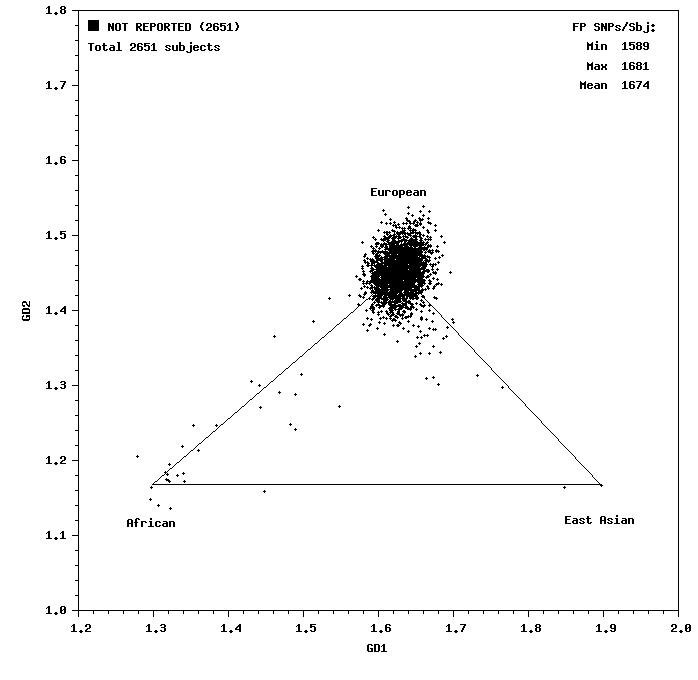

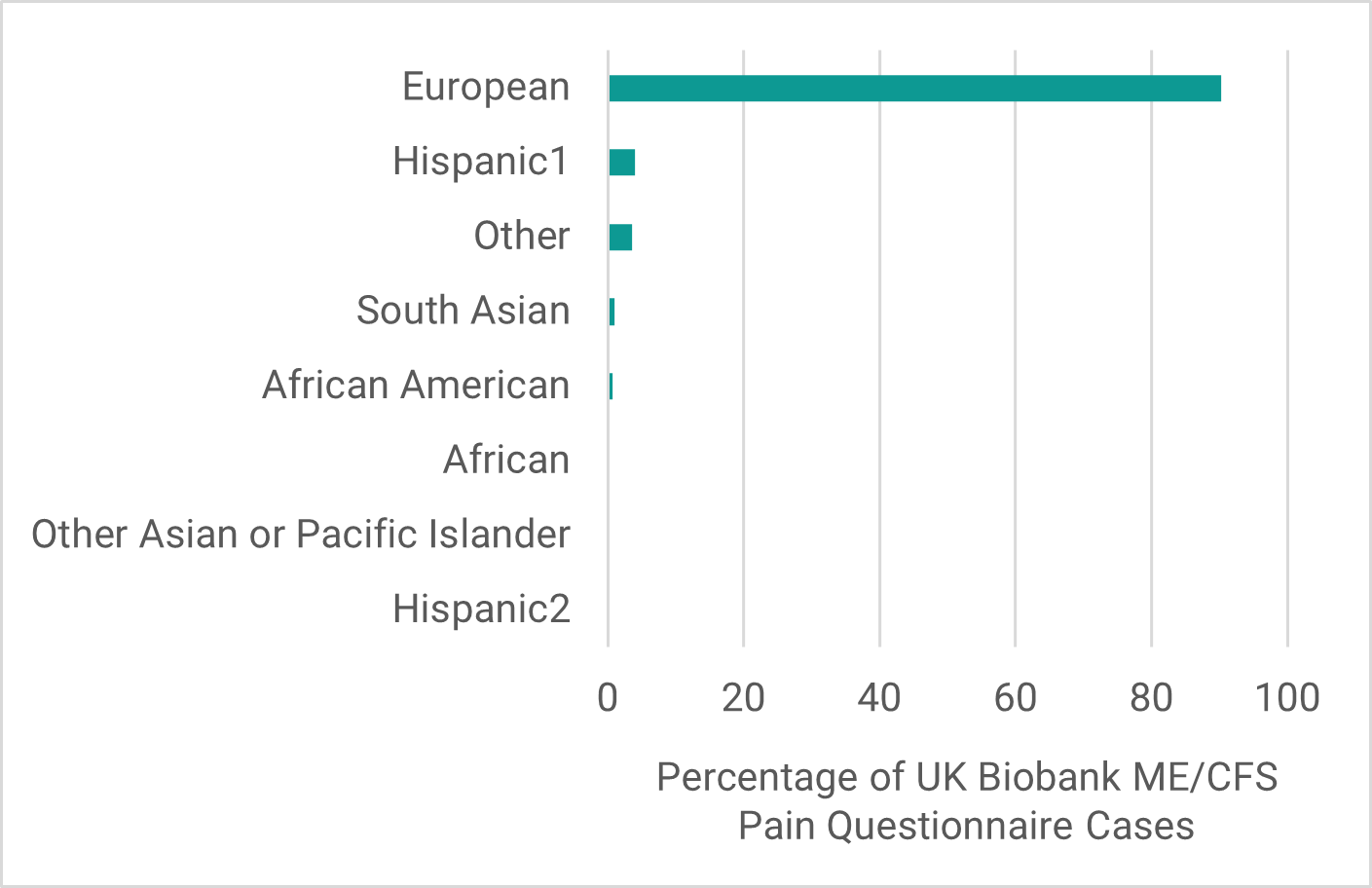


**(a) (b)**

Figure 6: (a) Ancestry inference plot generated by GRAF-pop and (b) the ancestry distribution of ME/CFS case population (n=2,651 cases) generated from UK-Biobank using self-reported diagnosis in Pain Questionnaire before quality control shows very strong bias for European ancestry (>90%).

## Cohort Analysis

### Data used for Cohort Analysis

Data used for cohort analysis in Figure 2:

### Exposure to infectious agents:

| Data Type | ICD-10/ Data Field /Category ID | Description |
| --- | --- | --- |
| Blood assays | 23053 | EBV seropositivity for Epstein-Barr Virus |
| Blood assays | 1307 | Infectious disease antigens |
| HES | A00.x-B99.x | Chapter I Certain infectious and parasitic diseases |

### Diagnosis with any of the most common autoimmune diseases:

| Data Type | ICD-10 / Data Field / Category ID | Description |
| --- | --- | --- |
| HES | M05.x or M06.x | Rheumatoid arthritis |
| HES | L40.x or M07.x | Psoriasis / psoriatic arthritis |
| HES | G35.x | Multiple sclerosis |
| HES | M32.x | Systemic lupus erythematosus |
| HES | K50.x or K51.x | Inflammatory bowel disease (Crohn’s and ulcerative colitis) |
| HES | K90.0 | Celiac disease |
| HES | M45.x | Ankylosing spondylitis |
| HES | M35.0 | Sjogren’s syndrome |
| HES | M35.3 | Polymyalgia rheumatica |
| HES | M34.x | Systemic sclerosis |
| HES | M79.7 | Fibromyalgia |
| HES | E10.x | Type 1 diabetes |
| HES | E06.x | Thyroiditis |

Evidence of significant stressful events:

| Data Type | ICD-10 / Data Field /Category ID | Description |
| --- | --- | --- |
| Self-reported | 6145 | Illness, injury, bereavement, stress in last two years (any but ‘none of the above’) |
| Self-reported | 20499 | Ever sought or received professional help for mental distress |
| Self-reported | 20500 | Ever suffered mental distress preventing usual activities |
| HES | Chapter XX | External causes of morbidity and mortality |

### Sex


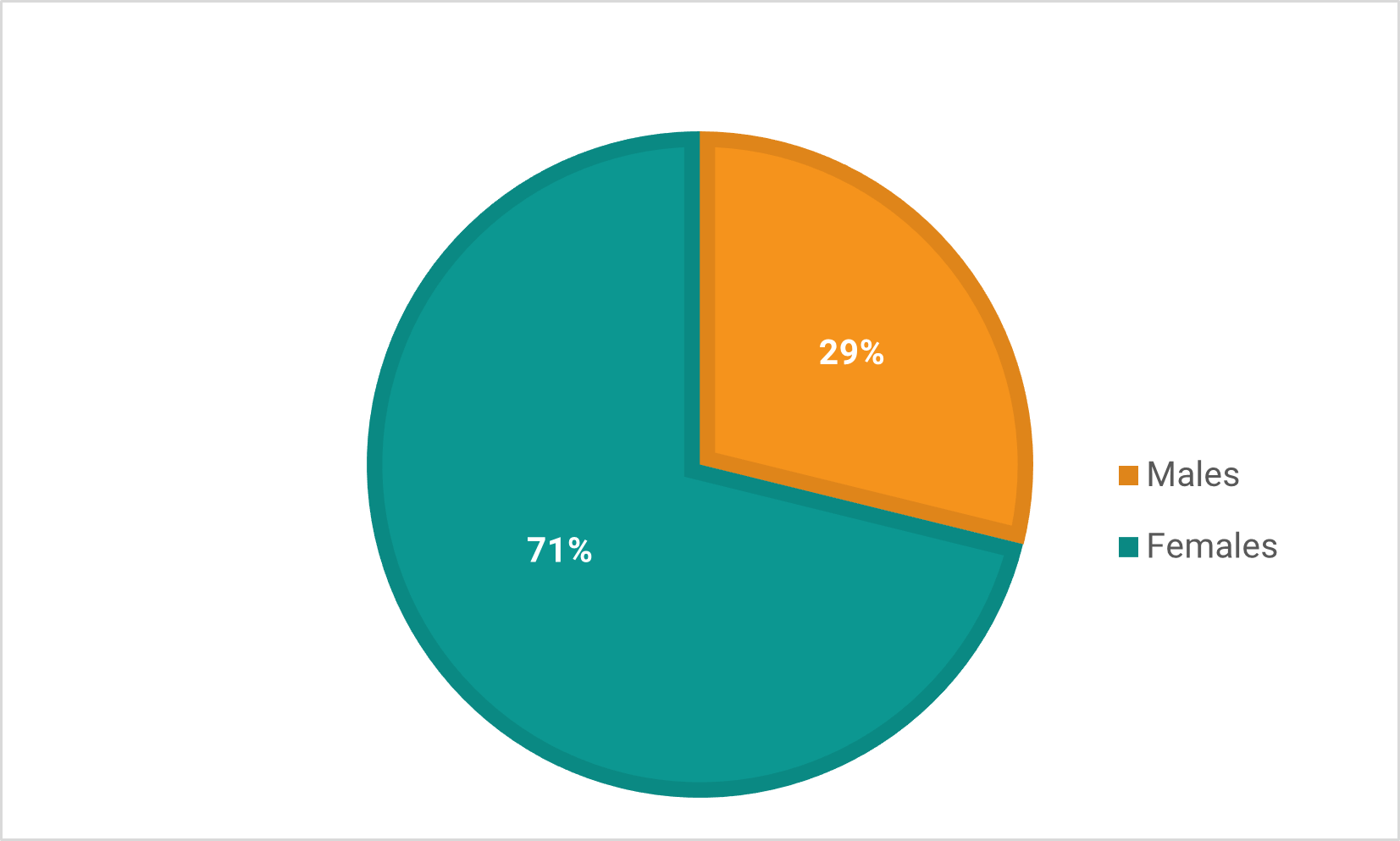


**Figure 7:** The proportion of females in the Pain Questionnaire case population was substantially higher (~71%) than males (29%).

### Age & BMI


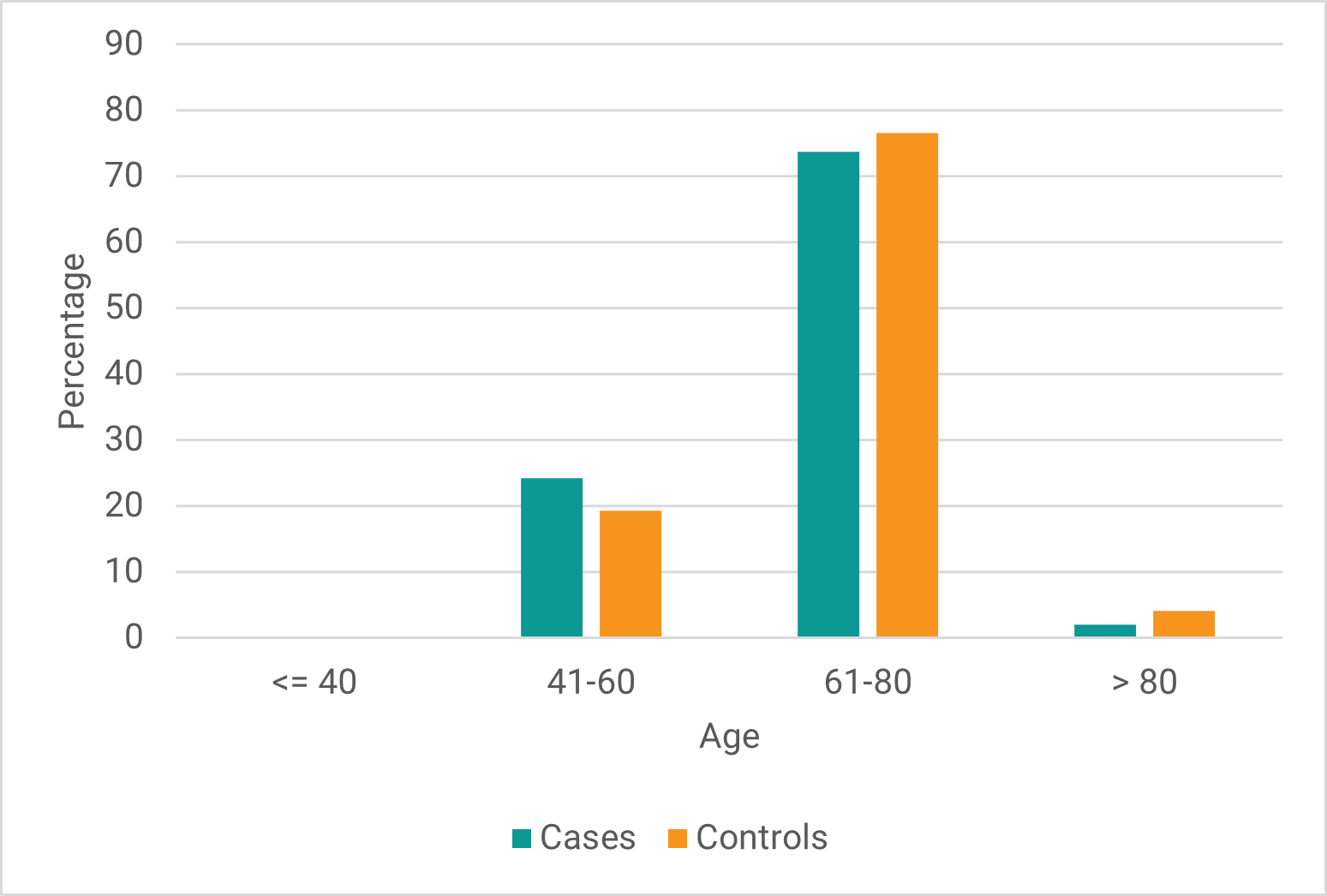

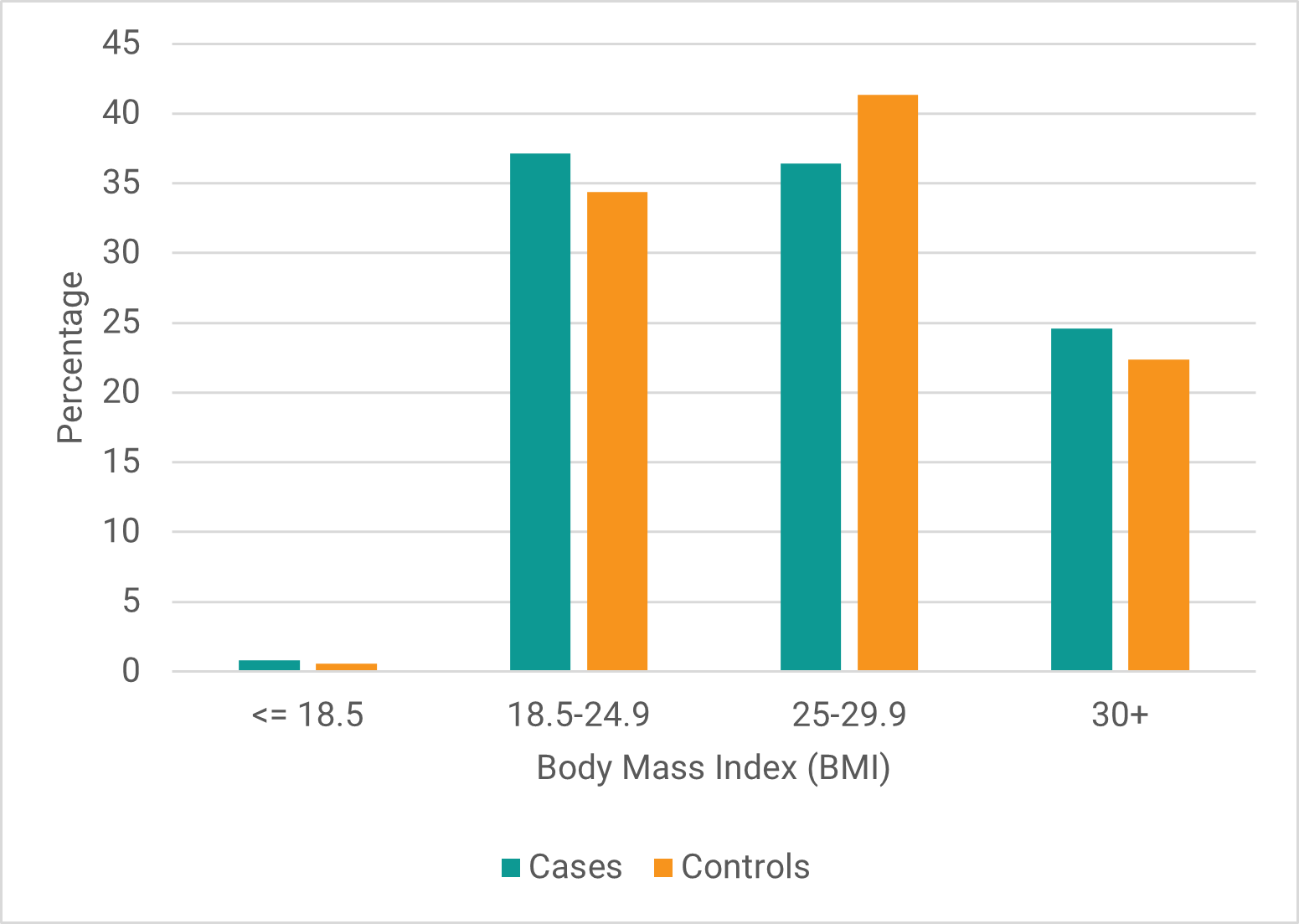


**(a) (b)**

Figure 8: Distribution of (a) age and (b) BMI of cases vs controls in the Pain Questionnaire cohort.

## GWAS Analysis
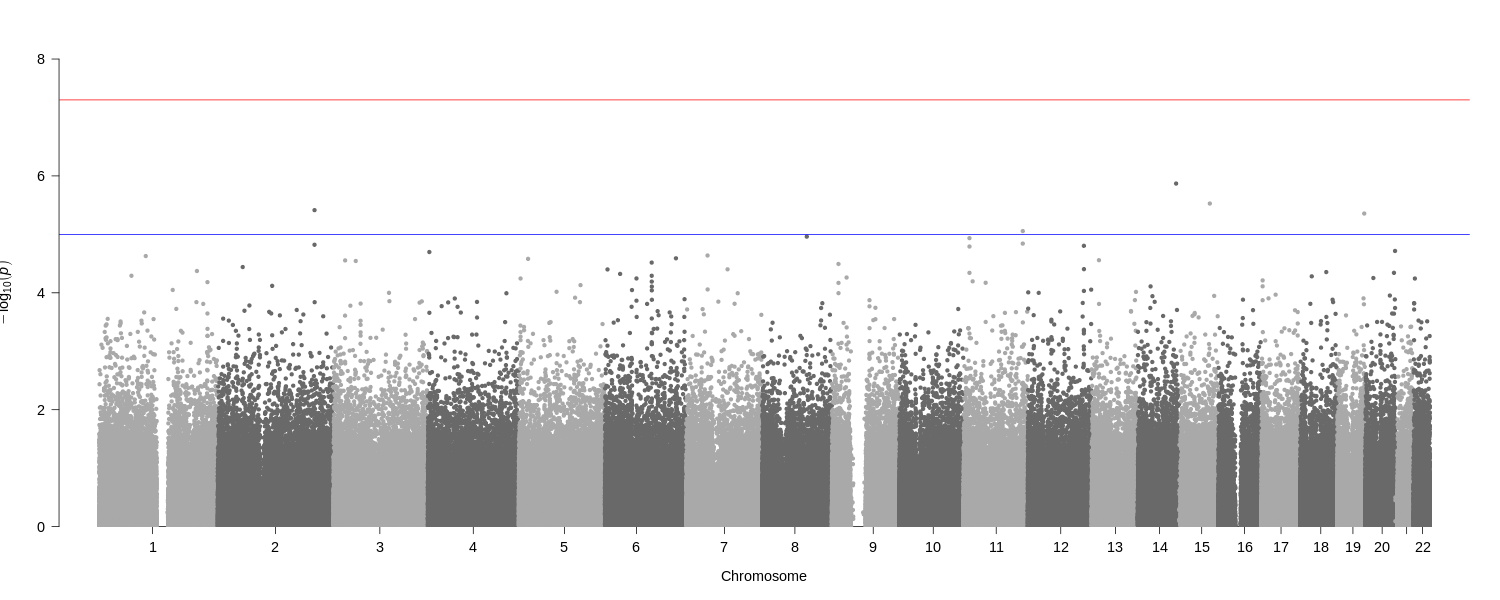


(a)


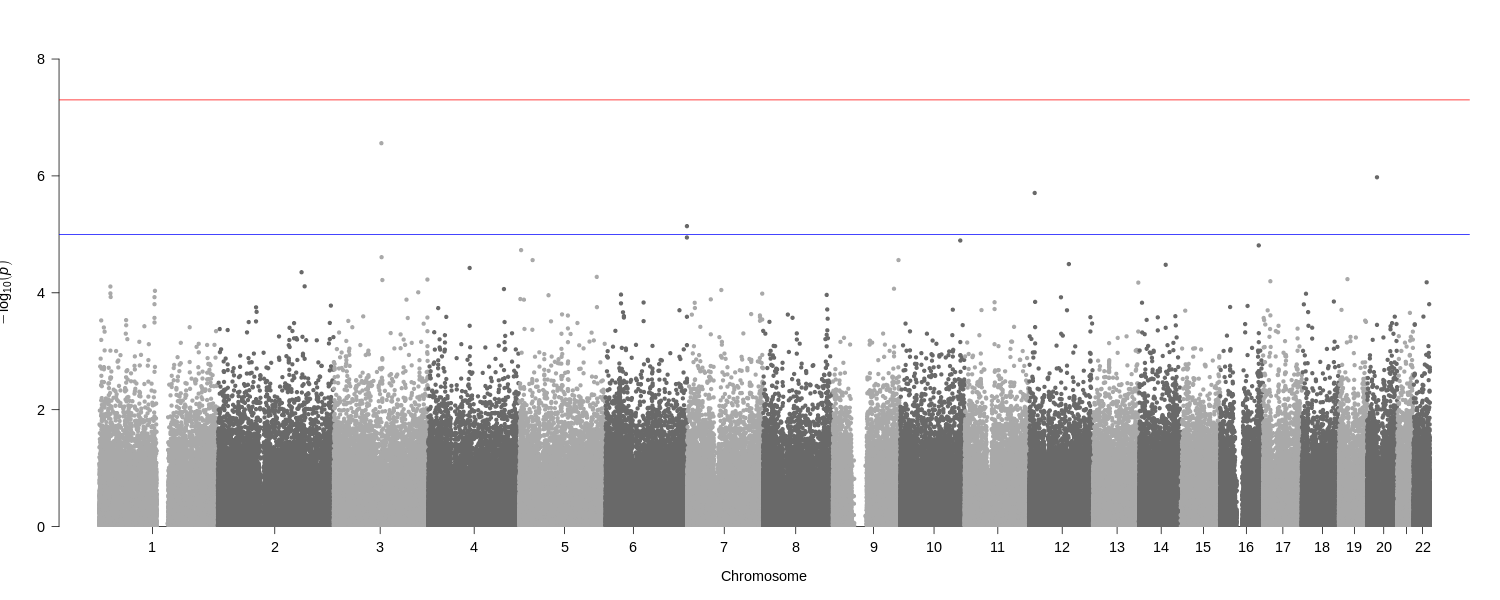


(b)

Figure 9: Manhattan plots generated using PLINK of genome-wide *p*-values of association for the (a) Pain Questionnaire cohort (n = 7,146 where cases=2,382 and controls=4,764) and (b) disjoint Verbal Interview cohort (n = 5,140 where cases=1,273 and controls=4,137). The horizontal blue and red lines represent the genome-wide significance values of *p*<1 x 10^-5^ and *p*<5 x 10^-8^ respectively.

## Combinatorial Disease Signatures

Table 6: Summary of thresholds used in the mining phase of the PrecisionLife combinatorial analysis on the Pain Questionnaire cohort.

| Layer | Minimum Z-Score | Minimum Cases (%) | Minimum Risk Ratio | No. of Reported Disease Signatures |
| --- | --- | --- | --- | --- |
| 1 (singleton) | 1.96 | 5% | 2.00 | 0 |
| 2 (pair) | 2.85 | 5% | 2.00 | 184 |
| 3 (triple) | 3.83 | 5% | 2.34 | 45,711 |
| 4 (quadruple) | 4.21 | 5% | 3.10 | 49,894 |
| 5 (quintuple) | 4.33 | 5% | 3.97 | 44,426 |

Table 7: List of 84 disease signatures with its constituent SNPs and mapped genes identified in the ME/CFS Pain Questionnaire study.

| Disease signature | SNPs | Genes |
| --- | --- | --- |
| Disease signature 1 | rs7731560, rs237475, rs2904106 | AL035685.1, KCNB1, ATP9A |
| Disease signature 2 | rs11695478, rs7837321, rs62112949 | TRAPPC9, LINC00683 |
| Disease signature 3 | rs11695478, rs7837321, rs66754206 | TRAPPC9, LINC00683 |
| Disease signature 4 | rs11695478, rs7837321, rs13047599 | TRAPPC9, SON |
| Disease signature 5 | rs11695478, rs10784508, rs4984526 | NR2F2-AS1, AC012409.2 |
| Disease signature 6 | rs10488419, rs2471534, rs2398428 | EXOC4, CEP290, SLC15A4 |
| Disease signature 7 | rs42756, rs77677798, rs2398428 | MCTP1, GTF3AP6, SLC15A4 |
| Disease signature 8 | rs7376617, rs4377693, rs60619978 | LINC02495, SCAMP1-AS1, KCTD1 |
| Disease signature 9 | rs76621327, rs7376617, Affx-34417209 | LINC02495, FBP1 |
| Disease signature 10 | rs7376617, rs28715644, rs5747306 | LINC02495, RAP1GAP2, BCL2L13 |
| Disease signature 11 | rs35537747, rs10177290, rs7376617 | LINC01789, AC005540.1, LINC02495 |
| Disease signature 12 | rs62401163, rs12530627, rs72732818 | CYP39A1, RPL36AP45 |
| Disease signature 13 | rs12530627, rs72732818, rs2309099 | RPL36AP45 |
| Disease signature 14 | rs11695478, rs58264436, rs7837321 | TMEM232, TRAPPC9 |
| Disease signature 15 | rs58264436, rs7007248, rs9646604 | TMEM232, ODF1, SOCS6 |
| Disease signature 16 | rs58264436, rs12309571, rs9646604 | TMEM232, SOCS6 |
| Disease signature 17 | rs2592394, rs58264436, rs7926655 | HOXD-AS2, TMEM232, SLC5A12 |
| Disease signature 18 | rs58264436, rs994779, rs7159803 | TMEM232, TTC6 |
| Disease signature 19 | rs72711020, rs58264436, rs9646604 | S100A2, TMEM232, SOCS6 |
| Disease signature 20 | rs58264436, rs4526126, rs10500403 | TMEM232, FAM153C, AC027130.1, IQCK |
| Disease signature 21 | rs58264436, rs4524616, rs7101688 | TMEM232, AL589740.1, OR52M1 |
| Disease signature 22 | rs7731560, rs62521813, rs237475, rs2904106 | C8orf34, AL035685.1, KCNB1, ATP9A |
| Disease signature 23 | rs7731560, rs11221492, rs237475, rs2904106 | AP000920.1, AL035685.1, KCNB1, ATP9A |
| Disease signature 24 | rs58634322, rs7731560, rs237475, rs2904106 | AC009486.1, AL035685.1, KCNB1, ATP9A |
| Disease signature 25 | rs57169846, rs11030639, rs16947237, rs4777865 | AC009468.1, STIM1, GPC5, AC091078.1 |
| Disease signature 26 | rs4738034, rs16947237, rs61744697, rs9979510 | AC079089.1, GPC5, DNAH9, AF212831.1 |
| Disease signature 27 | rs4738034, rs16947237, rs79935615, rs9979510 | AC079089.1, GPC5, DNAH9, AF212831.1 |
| Disease signature 28 | rs11030639, rs16947237, rs1956944, rs4777865 | STIM1, GPC5, OR7K1P, AC091078.1 |
| Disease signature 29 | rs11780906, rs7913215, rs16947237, rs6062914 | ADCY8, GDI2, GPC5, CHRNA4 |
| Disease signature 30 | rs13414838, rs17779800, rs16947237, rs351224 | NMS, GPC5, STRA6 |
| Disease signature 31 | rs13414838, rs9869834, rs16947237, rs351224 | NMS, SYNPR, SYNPR-AS1, GPC5, STRA6 |
| Disease signature 32 | rs12712033, rs9403525, rs12950480, rs3556 | KIAA1211L, PHACTR2, STX8, PEPD |
| Disease signature 33 | rs9403525, rs2028009, rs35463808, rs2244010 | PHACTR2, LINC02577, DPF3, AP000561.1 |
| Disease signature 34 | rs9403525, rs2028009, rs649372, rs35463808 | PHACTR2, LINC02577, SERTM1, DPF3 |
| Disease signature 35 | rs13138313, rs9403525, rs9299270, rs35774571 | AC108206.1, PHACTR2, AL441989.1, CBLN2 |
| Disease signature 36 | rs74135796, rs12332121, rs13285566, rs3785477 | AL450244.1, LINC02214, ZNF618, AKAP1 |
| Disease signature 37 | rs4844861, rs11135368, rs3785477, rs206604 | LAMB3, AC008415.1, AKAP1, AP005209.1 |
| Disease signature 38 | rs817095, rs1566129, rs3785477, rs10470164 | AC009975.2, AC009975.1, NID2, AKAP1, HUNK |
| Disease signature 39 | rs35388418, rs4601136, rs1445326, rs3785477 | AC097491.1, AL589740.1, AKAP1 |
| Disease signature 40 | rs2029952, rs3779859, rs7952262, rs35868427 | AC122138.1, MYOM2, NELL1, ETV6 |
| Disease signature 41 | rs1020411, rs2029952, rs3798276, rs7027070 | AC122138.1, TINAG, PAX5 |
| Disease signature 42 | rs2029952, rs17009338, rs72708234, rs684232 | AC122138.1, CCDC59, VPS53 |
| Disease signature 43 | rs2029952, rs17009338, rs684232, rs2190805 | AC122138.1, CCDC59, VPS53, AC006504.7, AC005357.2 |
| Disease signature 44 | rs2029952, rs17773312, rs684232, rs2190805 | AC122138.1, CCDC59, VPS53, AC006504.7, AC005357.2 |
| Disease signature 45 | rs60600282, rs2029952, rs12515588, rs12422918 | IGKV1OR2-108, AC122138.1, AC008581.2, ZBED3-AS1, AC022414.1, SRGAP1 |
| Disease signature 46 | rs7624046, rs6842554, rs2029952, rs11839732 | KCNMB2-AS1, KCNMB2, AC117457.1, AC097110.1, AC122138.1 |
| Disease signature 47 | rs41306603, rs58634322, rs7731560, rs237475, rs2904106 | S100PBP, AC009486.1, AL035685.1, KCNB1, ATP9A |
| Disease signature 48 | rs58634322, rs7731560, rs77821266, rs237475, rs2904106 | AC009486.1, AP001978.1, AL035685.1, KCNB1, ATP9A |
| Disease signature 49 | rs1986655, rs34694271, rs2499908, rs73021223, rs1644757 | FAT4, USP6NL, CDON, CAMSAP3 |
| Disease signature 50 | rs17571877, rs56309153, rs6912599, rs2499908, rs2378865 | LINC02238, ATOH8, OFCC1, USP6NL, AL139353.1, HEATR5A |
| Disease signature 51 | rs56309153, rs35396333, rs6912599, rs2499908, rs2378865 | ATOH8, YTHDC2, OFCC1, USP6NL, AL139353.1, HEATR5A |
| Disease signature 52 | rs56309153, rs35396333, rs6912599, rs2499908, rs7157977 | ATOH8, YTHDC2, OFCC1, USP6NL, AL139353.1, HEATR5A |
| Disease signature 53 | rs946448, rs7605428, Affx-33425392, rs73021223, rs6561713 | CYB5RL, AL357673.1, MRPL37, AC007402.1, FREM1, CDON, AL450423.1 |
| Disease signature 54 | rs12562306, rs7605428, Affx-33425392, rs73021223, rs6561713 | CYB5RL, AL357673.1, MRPL37, AC007402.1, FREM1, CDON, AL450423.1 |
| Disease signature 55 | rs72864990, rs9876108, rs6835113, rs209165, rs73021223 | FMNL2, LINC00882, ELOVL6, RPL13P, LINC01623, CDON |
| Disease signature 56 | rs946448, rs7605428, rs116395696, Affx-33425392, rs73021223 | CYB5RL, AL357673.1, MRPL37, AC007402.1, FREM1, CDON |
| Disease signature 57 | rs12562306, rs7605428, rs116395696, Affx-33425392, rs73021223 | CYB5RL, AL357673.1, MRPL37, AC007402.1, FREM1, CDON |
| Disease signature 58 | rs1267267, rs7930105, rs73021223, rs9304634, rs2042286 | MPLKIP, FBXO3, CDON, LINC00906 |
| Disease signature 59 | rs6861966, rs10884539, rs954090, rs4932370, rs59165976 | LINC01435, PTPRR, RN7SL363P, INSR |
| Disease signature 60 | rs10501233, rs7122851, rs2269787, rs4788817, rs59165976 | LRRC4C, AC090720.1, AP000446.1, SYT17, AC009097.2, INSR |
| Disease signature 61 | rs12044097, rs570817, rs10501233, rs6491316, rs59165976 | SPTA1, CFAP77, LRRC4C, AC090720.1, INSR |
| Disease signature 62 | rs61825301, rs35186300, rs570817, rs10501233, rs59165976 | OBSCN, CFAP77, LRRC4C, AC090720.1, INSR |
| Disease signature 63 | rs2304725, rs1395905, rs949730, rs10420798, Affx-16805420 | SLC6A11, AC027804.1, SYT1, AC090709.1, AC008738.6, SULF2 |
| Disease signature 64 | rs12490191, rs2304725, rs1395905, rs10420798, Affx-16805420 | SSUH2, SLC6A11, AC027804.1, AC008738.6, SULF2 |
| Disease signature 65 | rs12464809, rs62254551, rs6832769, rs9444564, rs4313118 | RN7SKP93, AC134729.1, CLOCK, TMEM165, AL590392.1, AC104370.1 |
| Disease signature 66 | rs2985754, rs726471, rs1327804, rs11839732, rs10403668 | NRCAM, TPT1P9, AP1M2 |
| Disease signature 67 | rs13015713, rs1917097, rs4745697, rs11839732, rs4813374 | SUCLA2P2, AC108752.1, RIN2 |
| Disease signature 68 | rs879665, rs1917097, rs10503277, rs519138, rs11839732 | MROH2A, AC108752.1, CSMD1, AC091096.1 |
| Disease signature 69 | rs4144251, rs56045138, rs17052708, rs11839732, rs11696277 | AC012368.1, LINC00445, PCSK2 |
| Disease signature 70 | rs73797049, rs11769606, rs28846183, rs12812478, rs11839732 | ABLIM2, ZNF767P, CASC8 |
| Disease signature 71 | rs2985754, rs726471, rs1327804, rs917602, rs11839732 | NRCAM, TPT1P9, PRMT8 |
| Disease signature 72 | rs817095, rs62452969, rs1566129, rs3785477, rs75827101 | AC009975.2, AC009975.1, AC007001.1, AC005062.1, NID2, AKAP1, LDLRAD4 |
| Disease signature 73 | rs74135796, rs2611215, rs758682, rs4501964, rs3785477 | AL450244.1, AC093655.1, TEAD1, AKAP1 |
| Disease signature 74 | rs12550751, rs4760980, rs12930636, rs3785477, rs11910362 | GPIHBP1, AC025252.1, AC093515.1, AKAP1 |
| Disease signature 75 | rs2236212, rs10764325, rs7953236, rs17136255, rs3785477 | ELOVL2, CACNB2, AXIN1, AKAP1 |
| Disease signature 76 | rs2236212, rs10764325, rs17136255, rs3785477, rs2243408 | ELOVL2, CACNB2, AXIN1, AKAP1 |
| Disease signature 77 | rs10018406, rs75548762, rs4601136, rs1445326, rs3785477 | AC097491.1, HMGN3, AL589740.1, AKAP1 |
| Disease signature 78 | rs10018406, rs4601136, rs116842259, rs1445326, rs3785477 | AC097491.1, AL589740.1, INSC, AKAP1 |
| Disease signature 79 | rs10011047, rs4601136, rs116842259, rs1445326, rs3785477 | AC097491.1, AL589740.1, INSC, AKAP1 |
| Disease signature 80 | rs2236212, rs55652946, rs10764325, rs17136255, rs3785477 | ELOVL2, CACNB2, AXIN1, AKAP1 |
| Disease signature 81 | rs17812116, rs10011047, rs4601136, rs1445326, rs3785477 | SUCLG2-AS1, AC097491.1, AL589740.1, AKAP1 |
| Disease signature 82 | rs4920832, rs62584791, rs7134469, rs3785477, rs8107775 | RPS2P25, AKAP1, R3HDM4 |
| Disease signature 83 | rs35388418, rs4601136, rs116842259, rs1445326, rs3785477 | AC097491.1, AL589740.1, INSC, AKAP1 |
| Disease signature 84 | rs62388082, rs2236212, rs10764325, rs17136255, rs3785477 | ELOVL2, CACNB2, AXIN1, AKAP1 |


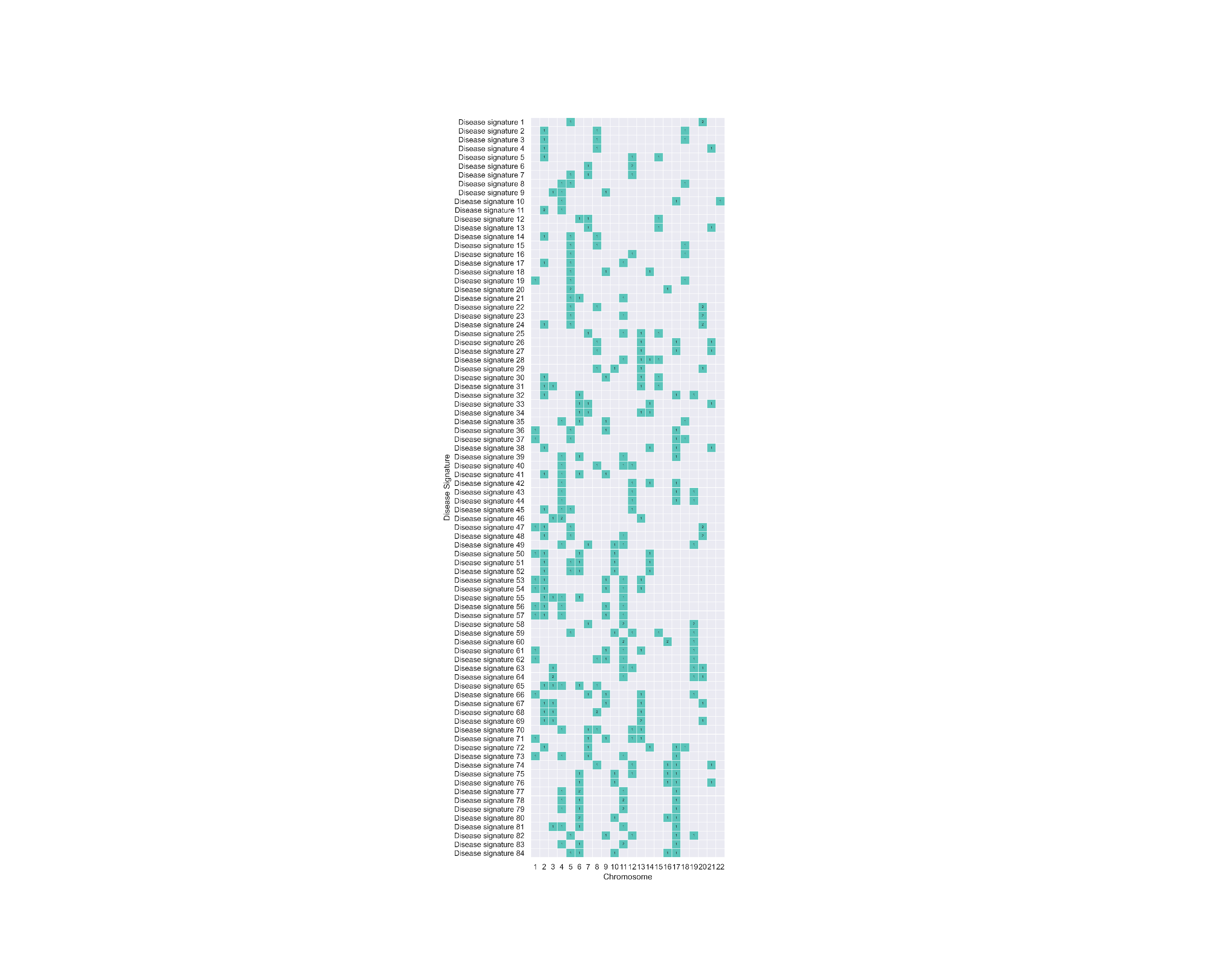


Figure 10: Distribution of chromosomal location of SNPs associated with 84 disease signatures identified in the Pain Questionnaire study. None of the SNPs identified in the disease signatures were observed to be in linkage disequilibrium (LD).


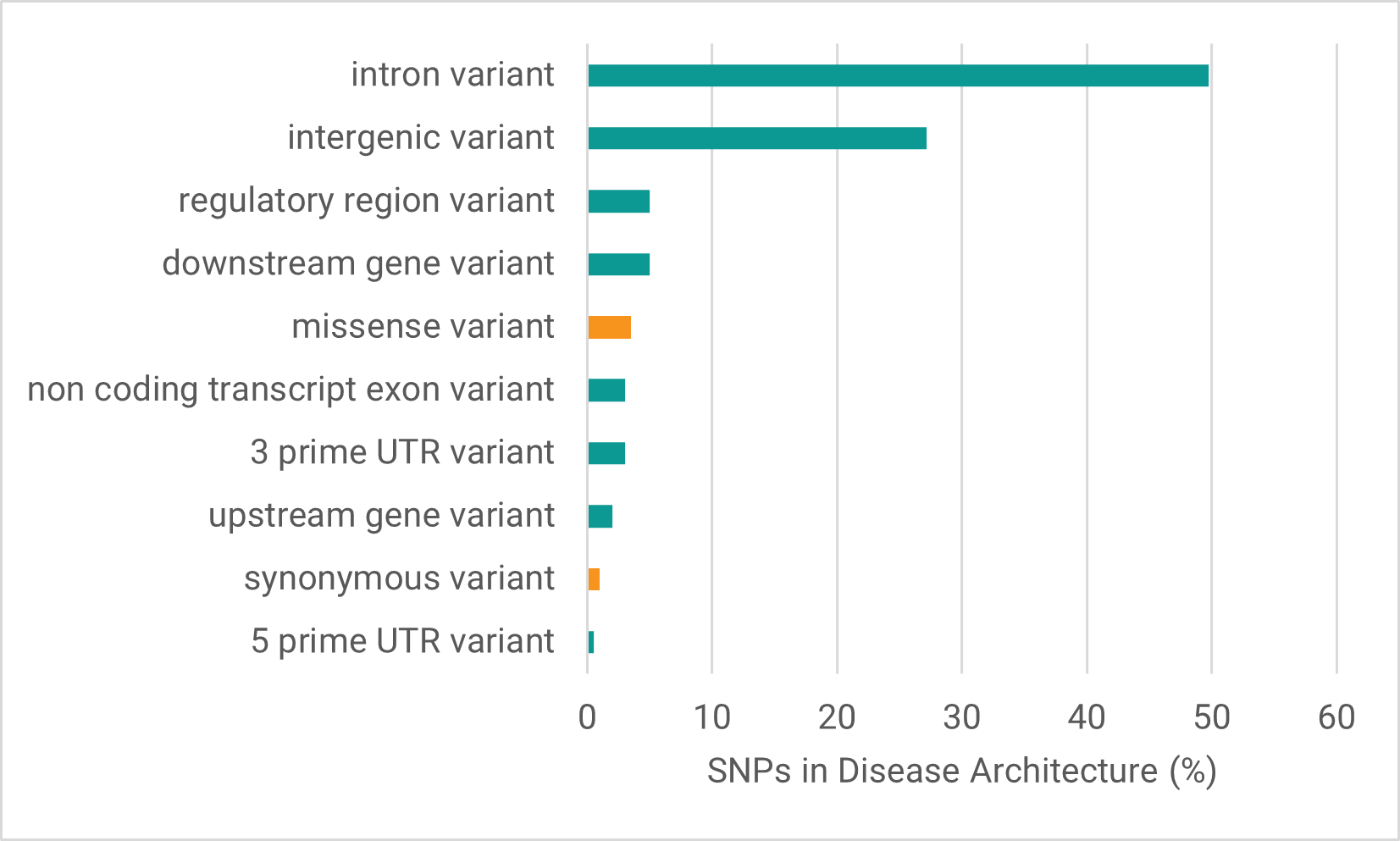


**Figure 11:** Distribution of variant consequences (most severe predicted by Ensembl VEP) of critical SNPs identified in the Pain Questionnaire study. More than 95% of SNPs were non-coding variants (shown in green) and <5% were coding variants (shown in orange).


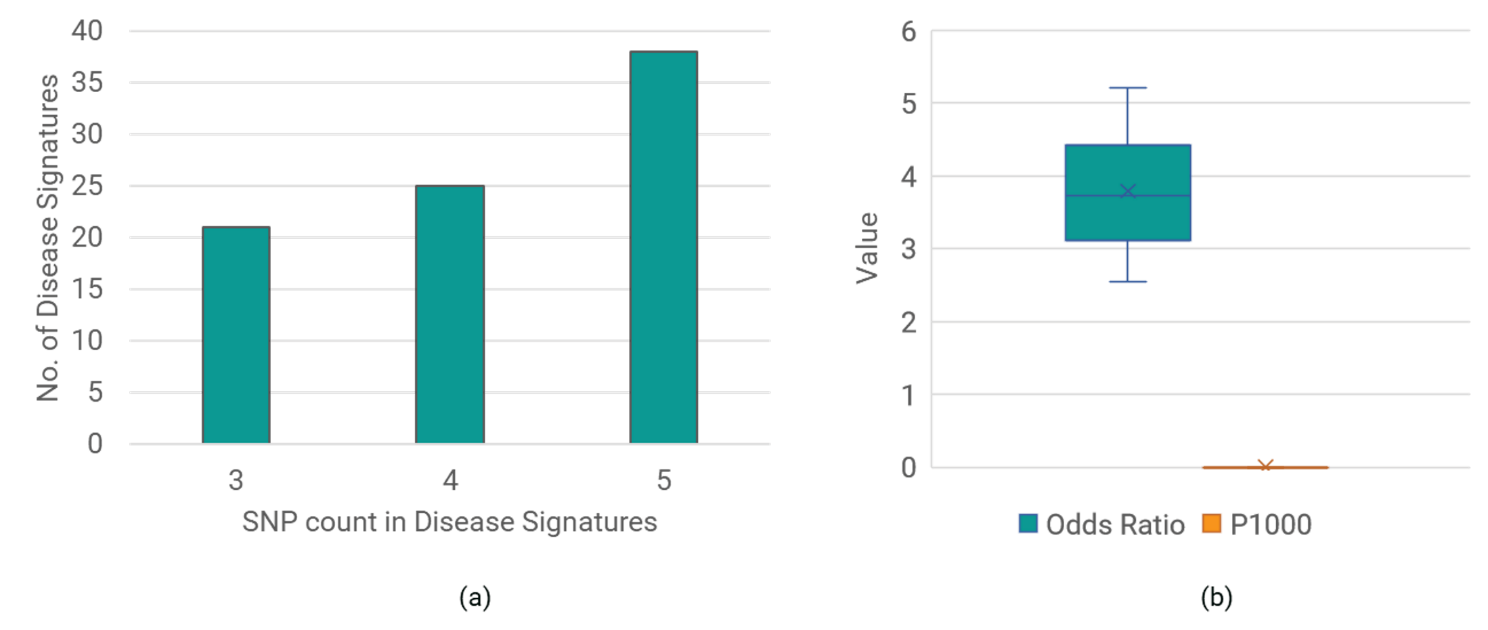


| SNP ID/Genotype | Mapped Genes | # Cases (t=2,382) | # Controls (t=4,764) |
| --- | --- | --- | --- |
| **rs58634322 / 0**  **rs7731560 / 2**  rs77821266 / 0  **rs237475 / 1**  **rs2904106 / 0** | **AC009486.1**  AP001978.1  **KCNB1/ AL035685.1**  **ATP9A** | 120 | 48 |

(c)

Figure 12: (a) Distribution of the combinatorial order of the 84 validated combinatorial disease signatures identified in the Pain Questionnaire cohort – i.e., 3 = signatures containing 3 co-associated SNPs. (b) Boxplot showing distribution of odds ratio and P1000 associated with 84 disease signatures identified in the Pain Questionnaire cohort. (c) Example of one of the combinatorial disease signatures contributing to Community 1 identified by the PrecisionLife combinatorial analysis of the Pain Questionnaire cohort. Bold text indicates the critical (RF-scored) SNPs (and the genes to which they are mapped) in this signature.


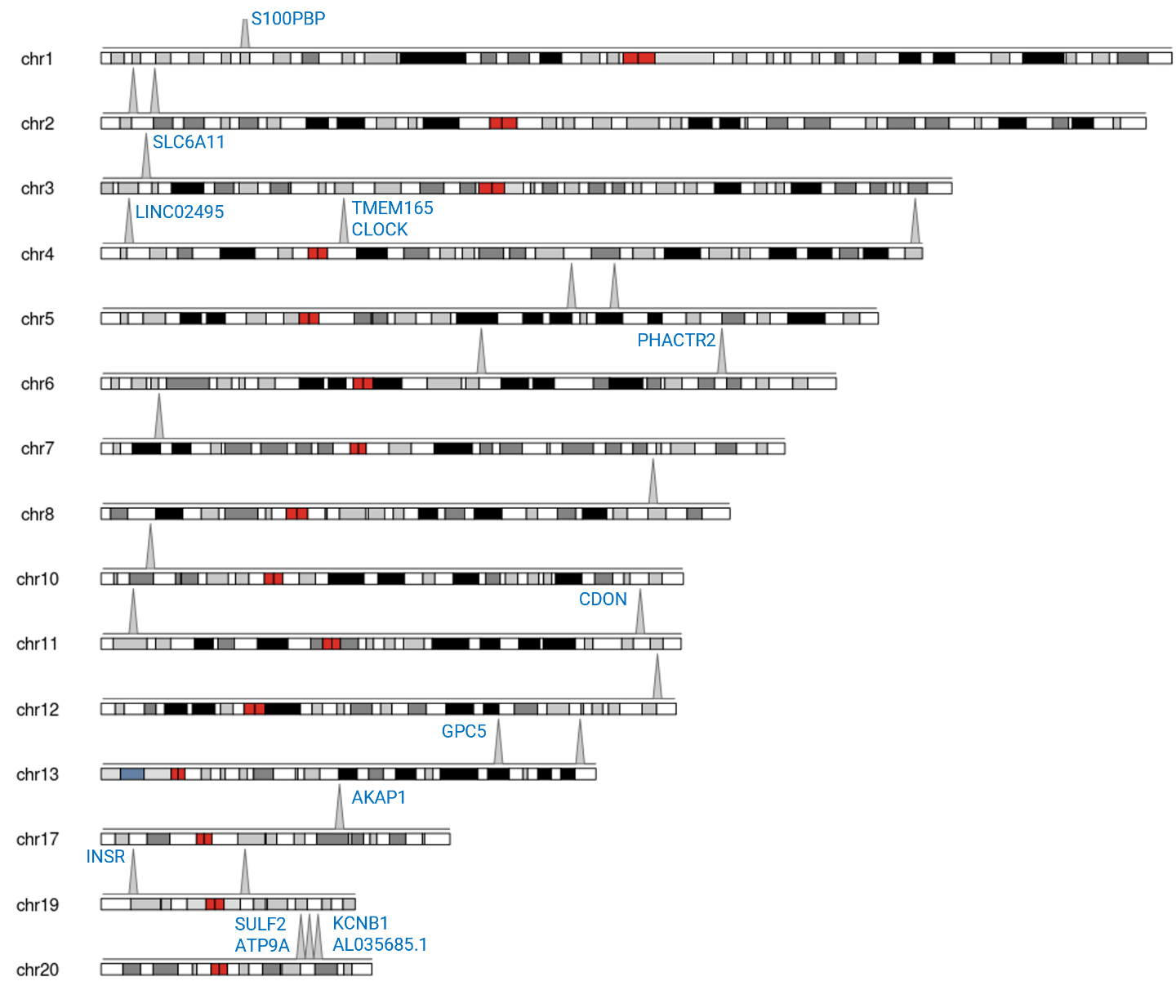


Figure 13: Graphical representation of the human autosomal chromosomes showing the locations (shown by peaks) for 25 critical disease-associated SNPs genes identified in the Pain Questionnaire cohort. The 14 disease-associated genes are annotated alongside the associated SNP in blue. The alternating light and dark regions in each chromosome represent bands along the length of the chromosome produced after staining with a dye such as Giemsa stain (G banding) and the red region represents the centromere.


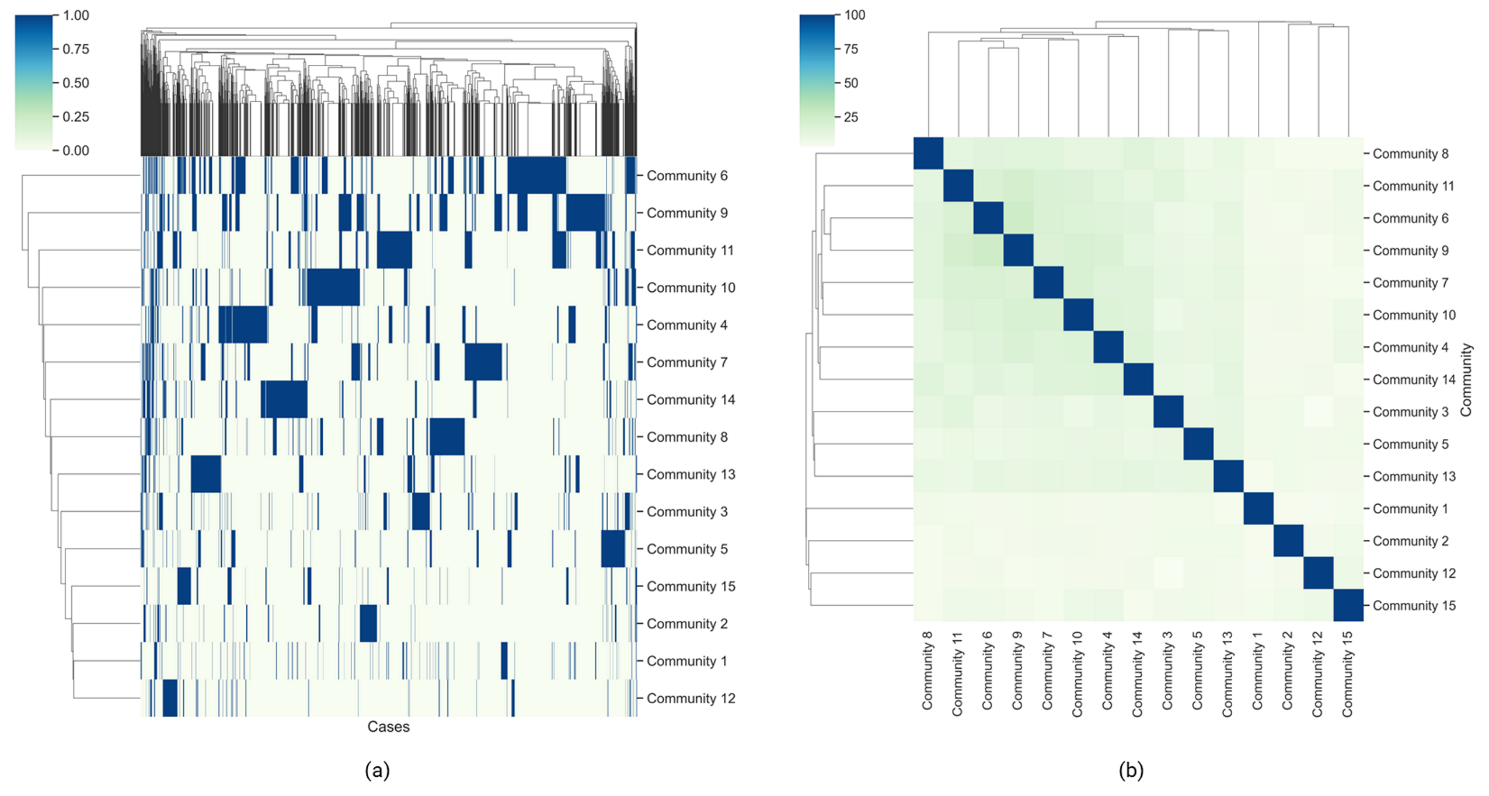
 Figure 14: (a) Clustered heatmap showing the similarity of the 15 communities based on their respective cohorts of associated Pain Questionnaire patients. (b) Clustered heatmap showing the 15 communities represent distinct patient sub-groups that share low <20% patient overlap with other communities.

Technical Replicate Study

As an internal validation we generated five smaller subsets of the Pain Questionnaire cohort each excluding a different 10% of the cases. These cross-validation runs therefore comprised 90% of the case population compared against the same set of controls as the Pain Questionnaire cohort. We compared the odds ratios and Z-scores of the disease signatures identified in the full cohort to those identified in each of the subset analyses. The odds ratios and Z-scores of the SNP combinations remain largely consistent irrespective of small changes to the case population (Figure 15).

This technical (internal cross-validation) replicate study provided an internal parameter check, but it has insufficient independence between both the case and control sets used in the runs for reliable use as formal replication.


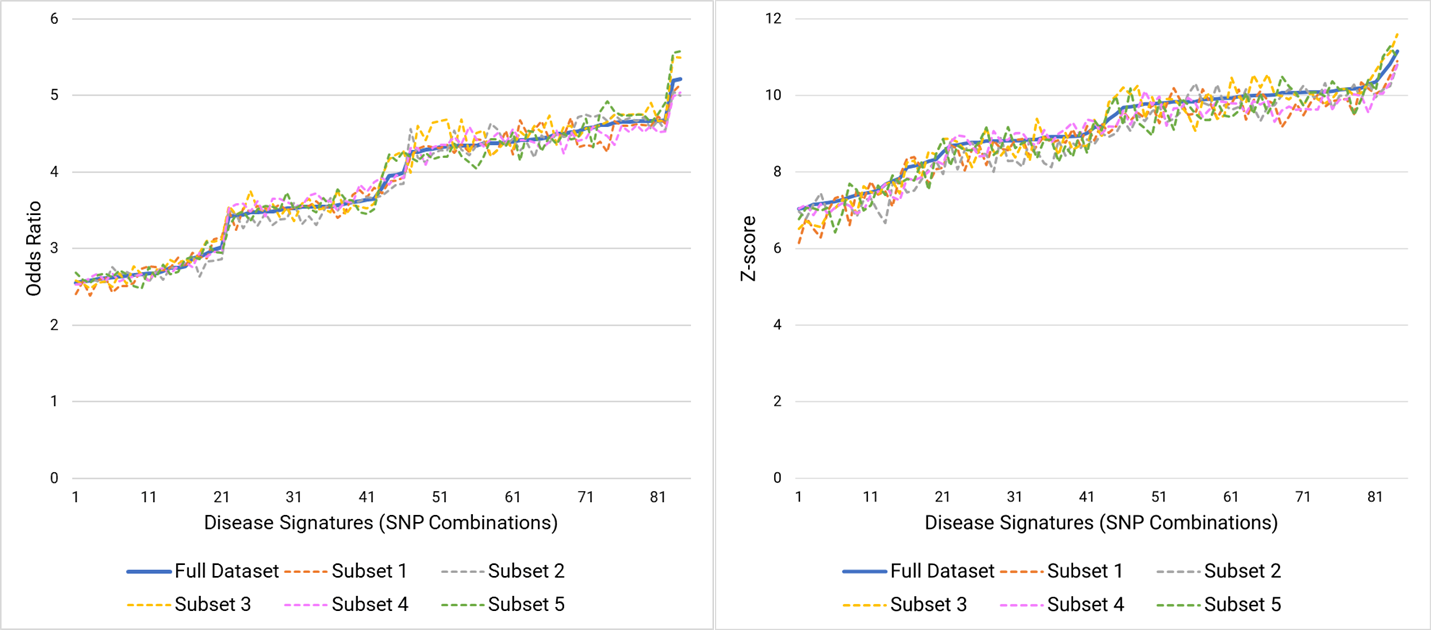


**(a) (b)**

Figure 15: Comparison of (a) odds ratios and (b) Z-scores of the full Pain Questionnaire dataset with five sub-cohort splits containing 90% of the same cases as the full cohort and the same set of controls.

Table 8: Disease signatures, SNPs and case count associated with communities identified in the Pain Questionnaire cohort.

| Community Number | Disease Signatures | SNPs | Case Count (t=2,382) | Control Count (t=4,764) | Chi Square  *p*-value | Odds Ratio |
| --- | --- | --- | --- | --- | --- | --- |
| 1 | 6 | 8 | 144 | 102 | 2.56 x 10^-17^ | 2.94 |
| 2 | 1 | 5 | 153 | 72 | 8.30 x 10^-29^ | 4.47 |
| 3 | 2 | 5 | 257 | 203 | 5.13 x 10^-26^ | 2.72 |
| 4 | 4 | 9 | 419 | 371 | 2.10 x 10^-35^ | 2.53 |
| 5 | 2 | 4 | 228 | 197 | 8.46 X 10^-20^ | 2.45 |
| 6 | 12 | 20 | 744 | 719 | 5.36 X 10^-57^ | 2.56 |
| 7 | 7 | 16 | 408 | 315 | 1.18 X 10^-43^ | 2.92 |
| 8 | 4 | 11 | 343 | 231 | 2.86 X 10^-44^ | 3.30 |
| 9 | 17 | 38 | 648 | 503 | 1.60 X 10^-72^ | 3.17 |
| 10 | 6 | 17 | 425 | 376 | 5.24 X 10^-36^ | 2.53 |
| 11 | 6 | 21 | 469 | 348 | 5.55 X 10^-54^ | 3.11 |
| 12 | 3 | 10 | 142 | 136 | 2.34 X 10^-10^ | 2.16 |
| 13 | 6 | 15 | 282 | 207 | 5.08 X 10^-32^ | 2.96 |
| 14 | 4 | 14 | 348 | 193 | 1.23 X 10^-56^ | 4.05 |
| 15 | 2 | 6 | 170 | 89 | 5.94 X 10^-29^ | 4.04 |

Genes Associated with Phenotypes

Table 9: Frequency distribution of categorical phenotypic features in cases associated with gene(s) compared to all cases and controls in the cohort. *p*-values were calculated to assess the association of each feature using two-sided Fisher’s exact tests.

| Phenotypic Feature | Gene(s) | Gene case count with feature | Gene Case count | All case count with feature | Total case count | All controls count with feature | Total control count | *p*-value (compared to cases) before multiple testing | Adjusted *p*-value (compared to cases) | *p*-value (compared to controls) before multiple testing | Adjusted *p*-value (compared to controls) |
| --- | --- | --- | --- | --- | --- | --- | --- | --- | --- | --- | --- |
| Illness injury bereavement stress in last 2 years (2019+) | *SLC6A11*  *SULF2* | 4 | 170 | 12 | 2,382 | 1 | 4,764 | 0.003 | 0.177 | 6.00 X 10^-21^ | 7.61 X 10^-21^ |
| Illness injury bereavement stress in last 2 years (2014+) | *CDON* | 48 | 328 | 249 | 2,382 | 3 | 4,764 | 0.023 | 0.861 | 6.36 X 10^-145^ | 1.19 X 10^-144^ |
| Fibromyalgia (ICD-10: M79.7) | *CLOCK* | 13 | 153 | 108 | 2,382 | 0 | 4,764 | 0.026 | 0.969 | 2.92 X 10^-90^ | 1.87 X 10^-89^ |
| Other soft tissue disorders (ICD-10: M79) | *CLOCK* | 22 | 153 | 224 | 2,382 | 0 | 4,764 | 0.044 | 0.969 | 1.15 X 10^-151^ | 1.04 X 10^-149^ |
| Thyroiditis (ICD10-10: E06) | *PHACTR2* | 3 | 343 | 5 | 2,382 | 0 | 4,764 | 0.033 | 0.969 | 1.07 X 10^-10^ | 1.92 X 10^-10^ |
| Males (Genetic sex) | *S100PBP* | 48 | 125 | 687 | 2,382 | 11 | 4,764 | 0.022 | 0.078 | 0 | 0 |
| Males (Genetic sex) | *ATP9A*  *KCNB1* | 55 | 144 | 687 | 2,382 | 31 | 4,764 | 0.016 | 0.078 | 7.29 X 10^-251^ | 3.28 X 10^-250^ |

Table 10: Frequency distribution of quantitative phenotypic features in cases associated with gene(s) compared to all cases and controls in the cohort. *p*-values were calculated to assess the association of each feature using two-sided Mann-Whitney U tests.

| Phenotypic Feature | Gene(s) | Gene case count with feature data | Max, Mean & Min value of feature for cases with gene assoc. | All case count with feature data | Max, Mean & Min value of feature for all cases | Control count with gene assoc. and feature data | Max, Mean & Min value of feature for controls | *p*-value (compared to cases) before multiple testing | Adjusted *p*-value (compared to cases) after multiple testing |
| --- | --- | --- | --- | --- | --- | --- | --- | --- | --- |
| Phenylalanine levels in plasma | *SLC6A11*  *SULF2* | 41 | 0.07  0.047 0.032 | 595 | 0.08 0.044 0.017 | 0 | N/A | 0.022 | 0.988 |

Table 11: Frequency distribution of quantitative phenotypic features in cases associated with communities compared to all cases and controls in the cohort. *p*-values were calculated to assess the association of each feature using two-sided Mann-Whitney U tests.

| Phenotypic Feature | Community | Gene case count with feature data | Max, Mean & Min value of feature for cases with gene assoc. | All case count with feature data | Max, Mean & Min value of feature for all cases | Control count with gene assoc. and feature data | Max, Mean & Min value of feature for controls | *p*-value (compared to cases) before multiple testing | Adjusted *p*-value (compared to cases) after multiple testing |
| --- | --- | --- | --- | --- | --- | --- | --- | --- | --- |
| Lactate levels in plasma | 3 | 61 | 1.81  3.41 6.81 | 595 | 1.41 3.69 9.64 | 0 | N/A | 0.028 | 0.992 |
| Lactate levels in plasma | 14 | 100 | 1.66  3.92 7.13 | 595 | 1.41 3.69 9.64 | 0 | N/A | 0.017 | 0.992 |

Genes Associated with SNPs using eQTL and Chromatin Interaction Data

**Table 12**: Gene assignments for SNPs using publicly available eQTL and chromatin-interaction data.

| SNP | Genes assigned using eQTL/chromatin data | Gene assignment approach | Tissue(s) of Interest | Genes assigned from genome assembly |
| --- | --- | --- | --- | --- |
| rs2904106 | *SALL4* | eqtl | Brain_Cerebellum | *ATP9A* |
| rs3785477 | *RP11-166P13.3* | eqtl | Whole_Blood,  Brain_Hypothalamus, Brain_Frontal_Cortex_BA9, Brain_Putamen_basal_ganglia,  Brain_Nucleus_accumbens_basal_ganglia | *AKAP1* |
| rs41306603 | *FNDC5* | eqtl | Brain_Frontal_Cortex_BA9 | *S100PBP* |
| rs6832769 | *SRD5A3* | eqtl | Whole_Blood, Brain_Cerebellum | *CLOCK* |
| rs11839732 | *LINC00346, TEX29* | promoter | Dorsolateral_Prefrontal_Cortex | - |

Tissue Expression of Genes


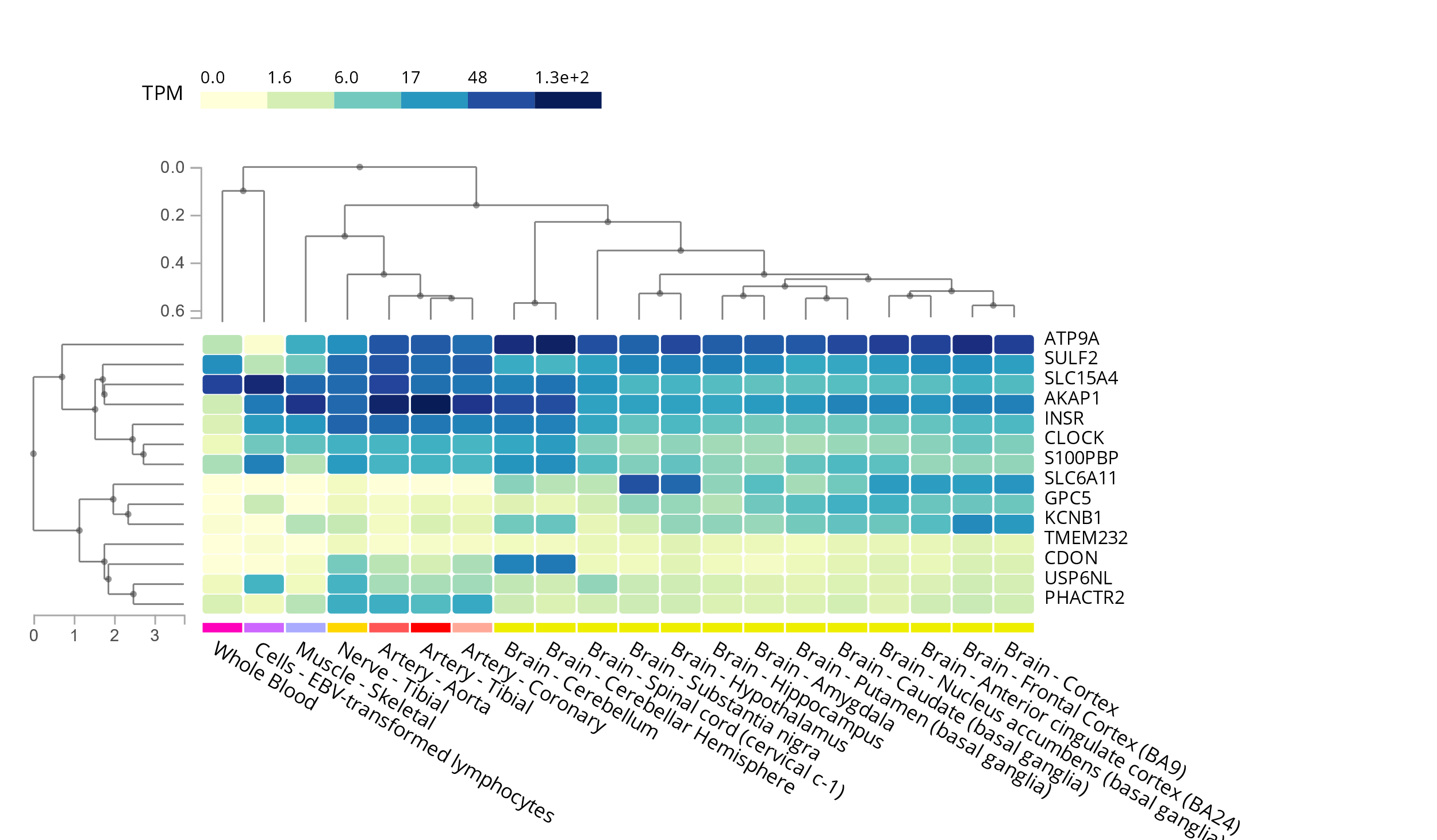


Figure 16: Clustered heatmap showing tissue expression profiles (GTEx) for 14 genes identified in the Pain Questionnaire study.

## Comparison with UK Biobank CFS Verbal Interview Study


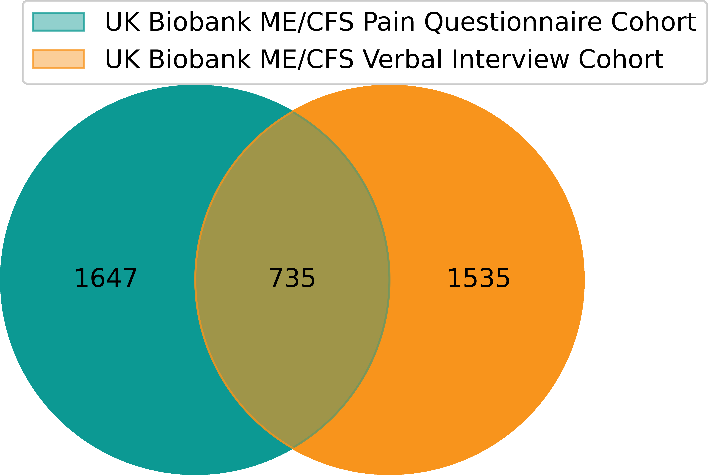


Figure 17: Case overlap between two UK Biobank ME/CFS cohorts (Pain Questionnaire and Verbal Interview)

## Gene Annotation Data Sources

Table 13: Example public annotation sources for various types of study results

| Annotation Type | Data Source(s) |
| --- | --- |
| **Encoded Protein Structure and Function** | Uniprot, InterPro, PDBe-KB |
| **SNP-Disease Association** | GWAS Catalog, PharmGKB, OpenTargets |
| **Gene-Disease Association** | PubMed, PubChem NCBI, OpenTargets |
| **Gene Tissue/Cell Expression** | Human Protein Atlas, GTEx, Promoter Capture HiC, Expression Atlas |
| **Pathways, Interactions or MoA** | Reactome, KEGG, Gene Ontology, PANTHER, WikiPathways, STRING, PubMed, IntAct |
| **Safety / Toxicology** | International Mouse Phenotyping Consortium, [Tox21](https://ntp.niehs.nih.gov/whatwestudy/tox21/index.html), eTox, HeCaToS, Open Targets |
| **Drugs and chemical compounds** | ChEMBL, DrugBank, PubChem NCBI, ProbeMiner, PDBe-KB |

## Pathway Enrichment Analysis

Pathway enrichment analysis was performed independently on all 199 disease signatures that included genes associated to any constituent SNP (critical or non-critical). The enrichment analysis was performed using gprofiler^140^ using Gene Ontology (excluding electronic annotations), KEGG and Reactome as data sources and considering only genes with at least one annotation as background genes. Only significant results (*p* < 0.05) after applying Bonferroni multiple testing correction were reported from the enrichment analysis.

Table 14: Biological pathway enrichment results for genes associated with disease signatures identified in the ME/CFS Pain Questionnaire study. Only significant results are reported from the enrichment analysis that used only non-electronic gene annotations, Bonferroni multiple testing correction and considered genes with at least one annotation as background genes.

| Disease Signature No. | Annotation source | Annotation name | Annotation ID | p value | Genes in Disease Signatures (query genes) | Genes with Annotation |
| --- | --- | --- | --- | --- | --- | --- |
| 1 | GO:BP | negative regulation of secretion by cell | GO:1903531 | 0.007028 | AL035685.1, KCNB1, ATP9A | KCNB1, ATP9A |
| 1 | GO:BP | negative regulation of secretion | GO:0051048 | 0.009192 | AL035685.1, KCNB1, ATP9A | KCNB1, ATP9A |
| 1 | GO:BP | regulation of exocytosis | GO:0017157 | 0.018417 | AL035685.1, KCNB1, ATP9A | KCNB1, ATP9A |
| 6 | WP | Ciliary landscape | WP:WP4352 | 0.003524 | EXOC4, CEP290, SLC15A4 | EXOC4, CEP290, SLC15A4 |
| 6 | REAC | Cilium Assembly | REAC:R-HSA-5617833 | 0.032864 | EXOC4, CEP290, SLC15A4 | EXOC4, CEP290, SLC15A4 |
| 22 | GO:BP | negative regulation of secretion by cell | GO:1903531 | 0.007028 | C8orf34, AL035685.1, KCNB1, ATP9A | C8orf34, KCNB1, ATP9A |
| 22 | GO:BP | negative regulation of secretion | GO:0051048 | 0.009192 | C8orf34, AL035685.1, KCNB1, ATP9A | C8orf34, KCNB1, ATP9A |
| 22 | GO:BP | regulation of exocytosis | GO:0017157 | 0.018417 | C8orf34, AL035685.1, KCNB1, ATP9A | C8orf34, KCNB1, ATP9A |
| 23 | GO:BP | negative regulation of secretion by cell | GO:1903531 | 0.007028 | AP000920.1, AL035685.1, KCNB1, ATP9A | KCNB1, ATP9A |
| 23 | GO:BP | negative regulation of secretion | GO:0051048 | 0.009192 | AP000920.1, AL035685.1, KCNB1, ATP9A | KCNB1, ATP9A |
| 23 | GO:BP | regulation of exocytosis | GO:0017157 | 0.018417 | AP000920.1, AL035685.1, KCNB1, ATP9A | KCNB1, ATP9A |
| 24 | GO:BP | negative regulation of secretion by cell | GO:1903531 | 0.007028 | AC009486.1, AL035685.1, KCNB1, ATP9A | KCNB1, ATP9A |
| 24 | GO:BP | negative regulation of secretion | GO:0051048 | 0.009192 | AC009486.1, AL035685.1, KCNB1, ATP9A | KCNB1, ATP9A |
| 24 | GO:BP | regulation of exocytosis | GO:0017157 | 0.018417 | AC009486.1, AL035685.1, KCNB1, ATP9A | KCNB1, ATP9A |
| 30 | REAC | Visual phototransduction | REAC:R-HSA-2187338 | 0.009099 | NMS, GPC5, STRA6 | NMS, GPC5, STRA6 |
| 31 | REAC | Visual phototransduction | REAC:R-HSA-2187338 | 0.009099 | NMS, SYNPR, SYNPR-AS1, GPC5, STRA6 | NMS, SYNPR, SYNPR-AS1, GPC5, STRA6 |
| 47 | GO:BP | negative regulation of secretion by cell | GO:1903531 | 0.007028 | S100PBP, AC009486.1, AL035685.1, KCNB1, ATP9A | S100PBP, KCNB1, ATP9A |
| 47 | GO:BP | negative regulation of secretion | GO:0051048 | 0.009192 | S100PBP, AC009486.1, AL035685.1, KCNB1, ATP9A | S100PBP, KCNB1, ATP9A |
| 47 | GO:BP | regulation of exocytosis | GO:0017157 | 0.018417 | S100PBP, AC009486.1, AL035685.1, KCNB1, ATP9A | S100PBP, KCNB1, ATP9A |
| 48 | GO:BP | negative regulation of secretion by cell | GO:1903531 | 0.007028 | AC009486.1, AP001978.1, AL035685.1, KCNB1, ATP9A | KCNB1, ATP9A |
| 48 | GO:BP | negative regulation of secretion | GO:0051048 | 0.009192 | AC009486.1, AP001978.1, AL035685.1, KCNB1, ATP9A | KCNB1, ATP9A |
| 48 | GO:BP | regulation of exocytosis | GO:0017157 | 0.018417 | AC009486.1, AP001978.1, AL035685.1, KCNB1, ATP9A | KCNB1, ATP9A |
| 49 | GO:BP | regulation of Golgi organization | GO:1903358 | 0.000946 | FAT4, USP6NL, CDON, CAMSAP3 | FAT4, USP6NL, CDON, CAMSAP3 |
| 59 | KEGG | MAPK signaling pathway | KEGG:04010 | 0.032815 | LINC01435, PTPRR, RN7SL363P, INSR | LINC01435, PTPRR, RN7SL363P, INSR |
| 60 | GO:BP | positive regulation of developmental growth | GO:0048639 | 0.035473 | LRRC4C, AC090720.1, AP000446.1, SYT17, AC009097.2, INSR | LRRC4C, SYT17, INSR |
| 63 | REAC | GABA synthesis, release, reuptake and degradation | REAC:R-HSA-888590 | 7.92E-05 | SLC6A11, AC027804.1, SYT1, AC090709.1, AC008738.6, SULF2 | SLC6A11, SYT1, SULF2 |
| 63 | KEGG | Synaptic vesicle cycle | KEGG:04721 | 0.000276 | SLC6A11, AC027804.1, SYT1, AC090709.1, AC008738.6, SULF2 | SLC6A11, SYT1, SULF2 |
| 63 | REAC | Neurotransmitter release cycle | REAC:R-HSA-112310 | 0.000634 | SLC6A11, AC027804.1, SYT1, AC090709.1, AC008738.6, SULF2 | SLC6A11, SYT1, SULF2 |
| 63 | REAC | Transmission across Chemical Synapses | REAC:R-HSA-112315 | 0.017284 | SLC6A11, AC027804.1, SYT1, AC090709.1, AC008738.6, SULF2 | SLC6A11, SYT1, SULF2 |
| 63 | REAC | Neuronal System | REAC:R-HSA-112316 | 0.041282 | SLC6A11, AC027804.1, SYT1, AC090709.1, AC008738.6, SULF2 | SLC6A11, SYT1, SULF2 |
